# Navigating the challenges of lateral ventricle epidermoid cysts: Diagnostic insights and surgical strategies. patient series

**DOI:** 10.1016/j.radcr.2025.05.087

**Published:** 2025-06-26

**Authors:** Moustafa A. Mansour, Saied A. Issa, Basim Ayoub, Moataz Abdelwahab

**Affiliations:** a Department of Neurosurgery, Nasser Institute for Research and Treatment, Cairo, Egypt; b Department of Neurosurgery, Faculty of Medicine, Helwan University, Cairo, Egypt; c Department of Neurosurgery, Faculty of Medicine, Cairo University, Cairo, Egypt; d Department of Neurosurgery, Kasr Al-Aini Hospital, Cairo, Egypt

**Keywords:** Epidermoid cyst, Lateral ventricle, Ventricular tumors, Microscopic-endoscopic surgery, CSF obstruction

## Abstract

Epidermoid cysts of the lateral ventricle are rare, benign intracranial lesions originating from ectopic embryonic epithelial cells, comprising only 0.2% of intracranial neoplasms. Although often asymptomatic initially, they may eventually cause mass effects or cerebrospinal fluid (CSF) obstruction as they enlarge. Accurate diagnosis—typically achieved via diffusion-weighted imaging (DWI)—is essential, as these cysts can mimic other intraventricular pathologies. Surgical resection remains the definitive treatment, though their deep-seated location and proximity to critical neurovascular structures pose significant challenges. This series describes 3 cases of lateral ventricle epidermoid cysts in patients presenting with progressive symptoms, including headaches, visual deficits, and neurological impairments, consistent with elevated intracranial pressure. Preoperative imaging, notably DWI, confirmed the diagnoses by demonstrating characteristic restricted diffusion. Near-total resection was achieved using microsurgical and endoscopic techniques, preserving vital structures and resulting in favorable postoperative outcomes with symptom resolution. However, 1 patient experienced persistent visual deficits due to delayed intervention. Advanced imaging, particularly DWI, is indispensable for precise diagnosis, while combined microscopic-endoscopic approaches optimize resection and reduce neurological risks. Early diagnosis and tailored surgical strategies are crucial for optimal outcomes. This series underscores the need for standardized management guidelines and identifies barriers to long-term follow-up, such as financial constraints.

## Introduction

Epidermoid cysts of the lateral ventricle are exceptionally rare, benign intracranial lesions arising from ectopic embryonic epithelial cells during neural tube closure [1]. Although epidermoid cysts account for only 0.2% of intracranial neoplasms, their occurrence in the lateral ventricle is even rarer compared to more frequent locations like the cerebellopontine angle or parasellar regions [2]. These slow-growing lesions often remain asymptomatic for years, with clinical manifestations—such as headaches, hemiparesis, seizures, or neuropsychological disturbances—emerging only when the cyst causes mass effect or CSF obstruction [[1], [2], [3]].

Accurate preoperative diagnosis relies heavily on magnetic resonance imaging (MRI), as these cysts can mimic other intraventricular pathologies, including meningiomas, ependymomas, or choroid plexus papillomas [1,4]. Complete surgical resection is the treatment of choice for symptomatic cases, though their deep-seated location demands specialized approaches, such as the interhemispheric transcallosal or transcortical routes [1,3]. Challenges arise from the cyst’s proximity to critical neurovascular structures and the risk of chemical meningitis from spilled contents. Notably, recurrence rates approach 33%, often due to incomplete resection [2].

In this case series, we present 3 patients with lateral ventricle epidermoid cysts who underwent successful total resection. These cases illustrate the diagnostic and surgical complexities of this rare entity while demonstrating favorable outcomes with meticulous management. By contributing to the sparse literature on this condition, we emphasize the importance of early diagnosis, tailored surgical planning, and long-term follow-up.

## Case presentation

### Case 1

A 23-year-old male presented to our neurosurgery unit with a 2-month history of worsening headaches and 1 month of complete blindness. His symptoms had begun 1 year earlier as intermittent headaches that progressed to frequent, throbbing pain severe enough to disrupt daily activities. He reported associated early-morning vomiting that provided temporary headache relief, along with developing somnolence and episodes of irrational speech. Over time, he experienced progressive right-sided weakness affecting his mobility and gradual vision loss culminating in total blindness 1 month before presentation. There was no history of seizures, head trauma, radiation exposure, tuberculosis symptoms, hypertension, diabetes, or known drug allergies.

On examination, the patient was alert with stable vital signs and a Glasgow Coma Scale (GCS) score of 15. Visual assessment showed no light perception in either eye, with moderately dilated, nonreactive pupils and blurred optic disc margins bilaterally on fundoscopy. Motor examination revealed right hemiparesis, more pronounced in the upper limb, with hyperreflexia in both knees. The remainder of the systemic examination was normal. Based on these findings, a provisional diagnosis of a space-occupying lesion causing blindness secondary to elevated intracranial pressure was made.

**Fig. 1:**
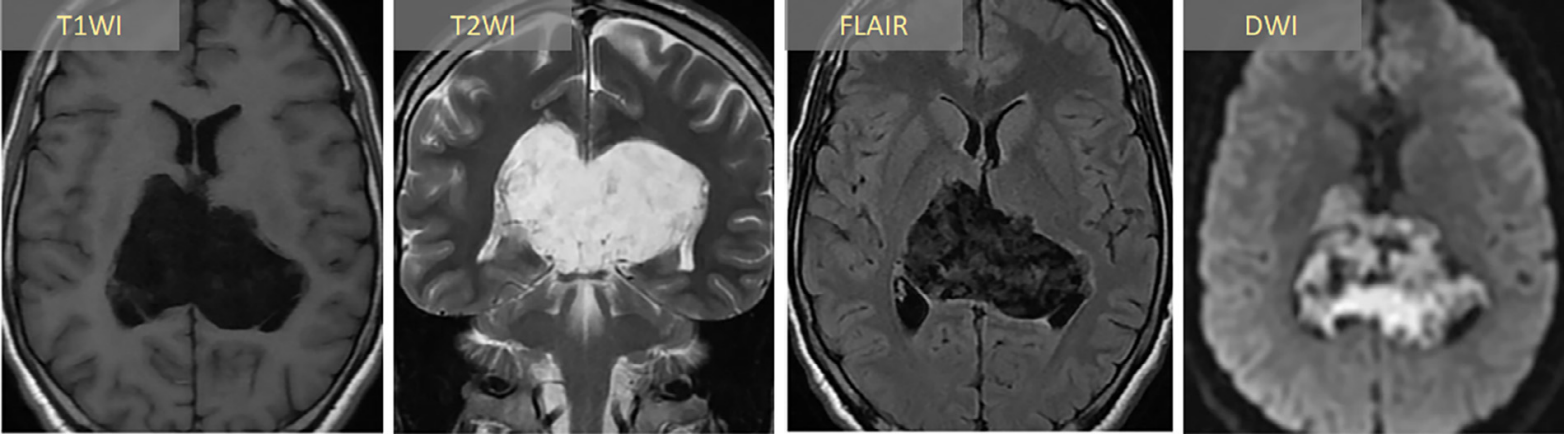
Axial MRI sequences demonstrate a large biventricular epidermoid tumor with posterior extension to the pineal region. The lesion exhibits: (A) heterogeneous hypointensity on T1WI; (B) hyperintensity on T2WI; (C) incomplete signal suppression on FLAIR; and (D) marked diffusion restriction on DWI. *T1WI: T1-weighted imaging; T2WI: T2-weighted imaging; FLAIR: fluid-attenuated inversion recovery; DWI: diffusion-weighted imaging.*

**Fig. 2:**
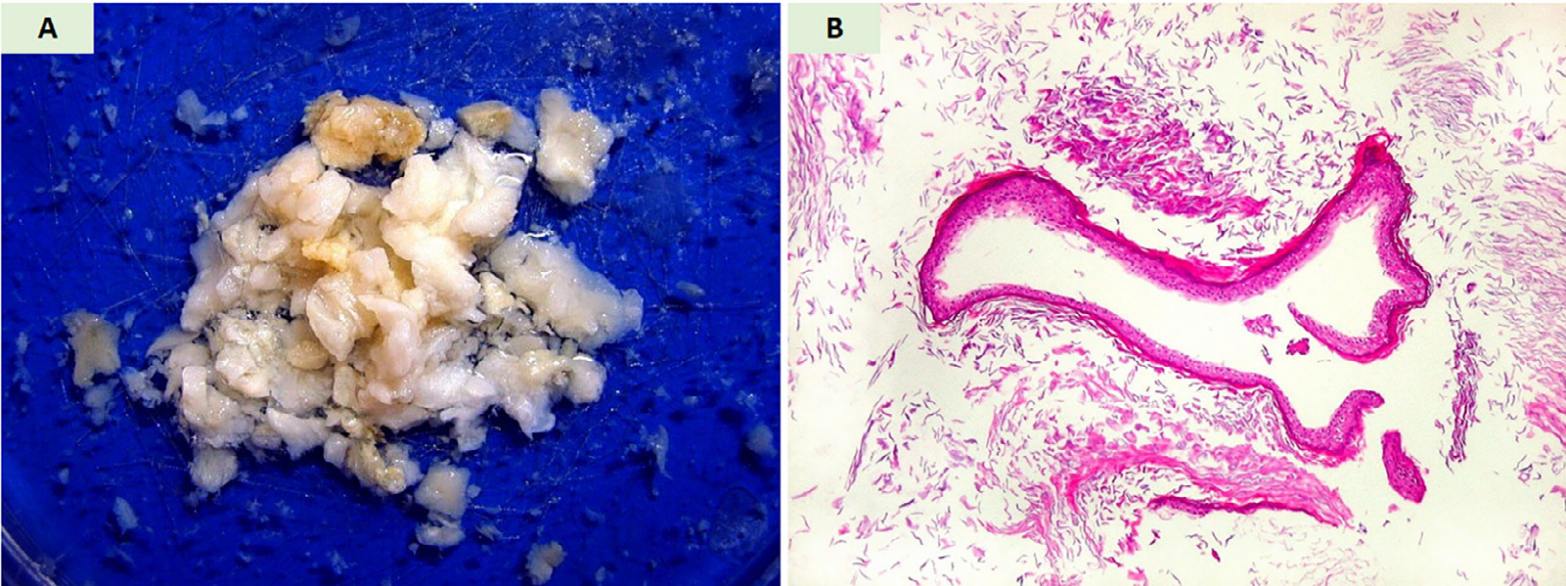
Histopathological characteristics of the resected epidermoid cyst. (A) Gross specimen showing friable, flaky fragments with variable coloration (light cream to brownish) that floated in fixative solution, suggesting low density secondary to high lipid content. (B) Hematoxylin and eosin (H&E) stained sections revealing: (i) laminated keratinous debris (non-nucleated corneal lamellae); (ii) rare fragments of stratified keratinized epithelium from the cyst lining; and (iii) the characteristic growth pattern whereby epithelial desquamation leads to gradual cystic expansion and adjacent structure compression.

Brain MRI demonstrated a large, well-encapsulated mass arising from the occipital horns of the lateral ventricles. The lesion exhibited both cystic and solid components with diffusion restriction and incomplete FLAIR suppression (Fig. 1). Following counseling and written consent, the patient underwent craniotomy with tumor excision via posterior callosotomy. The lateral tumor extensions posed a surgical challenge due to their location in the surgeon's blind spot, necessitating endoscopic assistance for complete visualization and removal. Intraoperatively, the tumor had a soft, cheesy consistency and was completely excised by simple scooping. Histopathology confirmed an epidermoid cyst (Fig. 2).

**Fig. 3:**
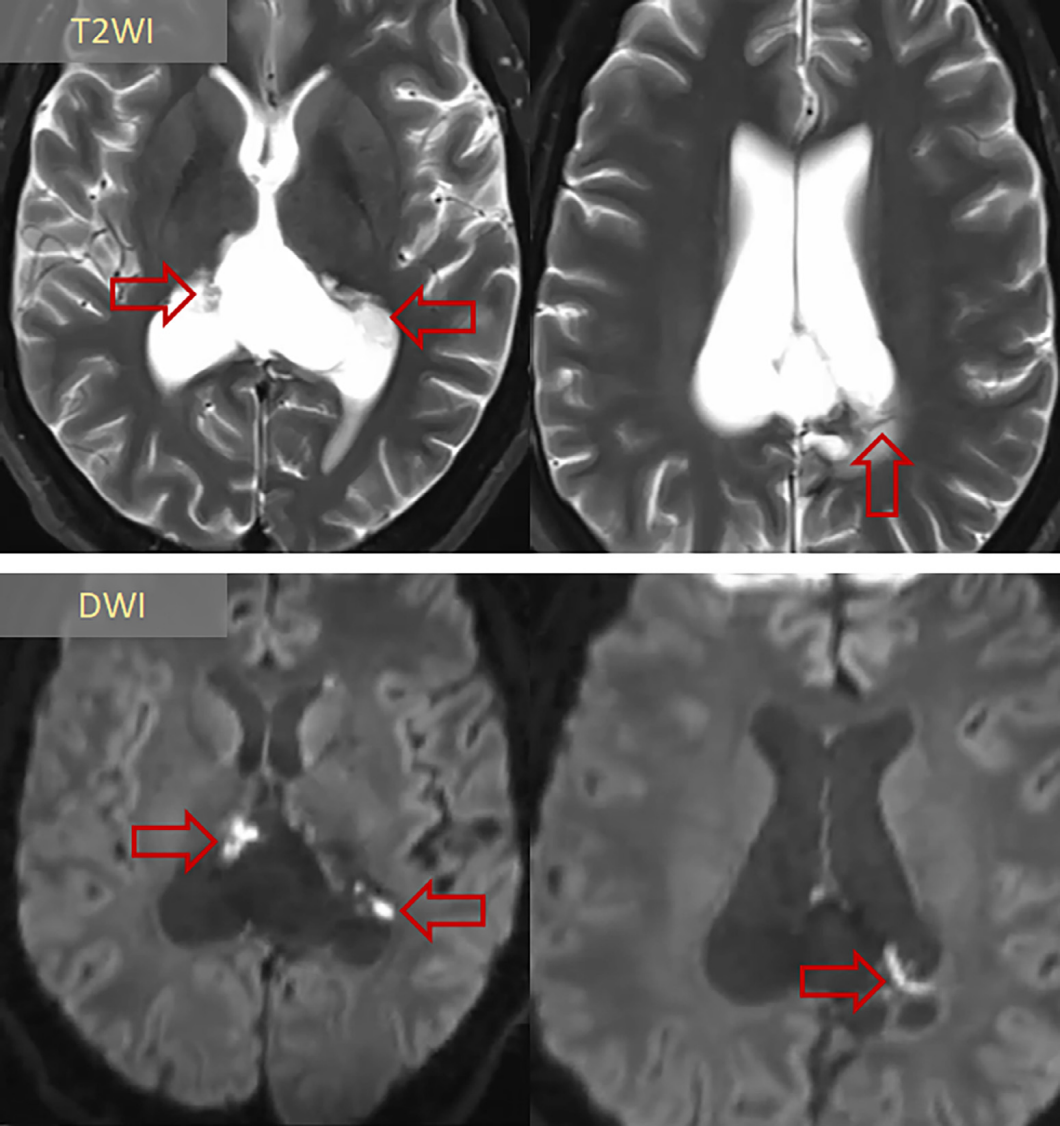
Three-month postoperative MRI surveillance following near-total resection (>95% tumor volume reduction). (A) T2WI and (B) DWI demonstrate minimal residual tumor remnants (*arrows*) deliberately preserved beneath the ependymal layer to prevent neurological morbidity, with no evidence of postoperative complications. *T2WI: T2-weighted imaging; DWI: diffusion-weighted imaging.*

Postoperatively, the patient recovered well and was discharged. At his 3-month follow-up, repeat MRI showed near-total (>95%) resection without complications (Fig. 3). While his blindness persisted, all symptoms of elevated intracranial pressure had resolved completely.

### Case 2

A 34-year-old male presented with a 5-year history of recurrent headaches that had dramatically worsened over the preceding 2 months, culminating in a severe episode that prompted emergency evaluation. Initial CT imaging revealed a low-density lateral ventricular lesion. Over the previous 2 years, the patient had developed progressive memory impairment and reduced responsiveness, though neurological examination remained unremarkable.

**Fig. 4:**
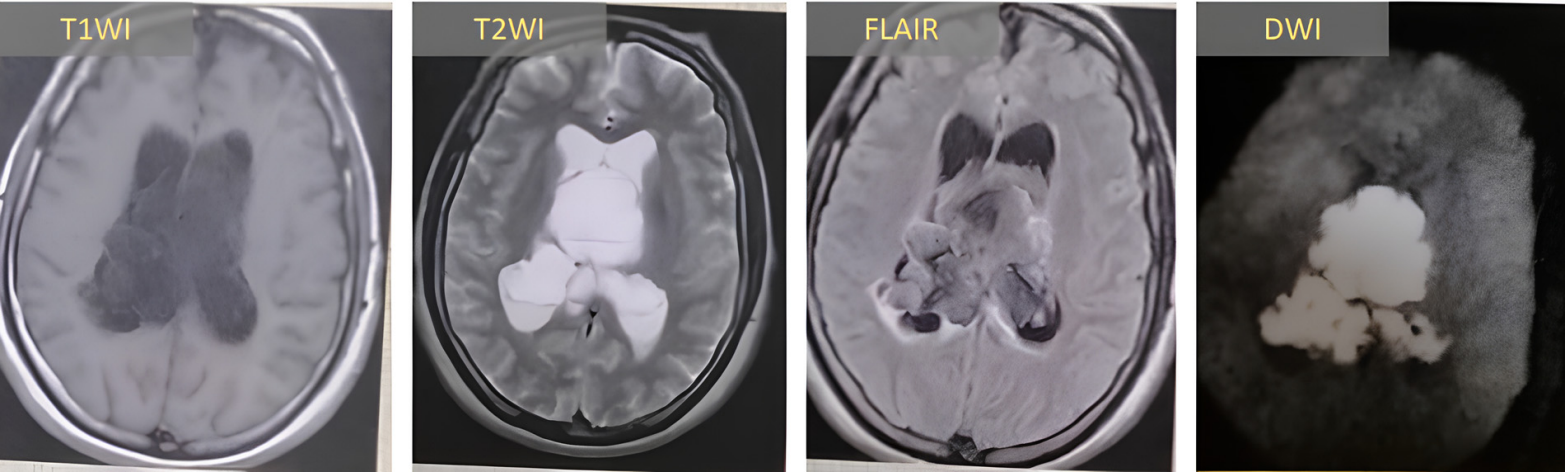
Preoperative brain MRI of a multiloculated intraventricular epidermoid cyst. The lesion displays: (A) hypointense signal on T1WI; (B) hyperintense signal on T2WI; (C) incomplete nulling on FLAIR; and (D) restricted diffusion on DWI. Note the mass effect with hypothalamic compression, suprasellar cistern effacement, and near-complete obliteration of the mammillo-pontine space. *T1WI: T1-weighted imaging; T2WI: T2-weighted imaging; FLAIR: fluid-attenuated inversion recovery; DWI: diffusion-weighted imaging.*

Advanced MRI characterization showed a large, multiloculated lesion with smooth margins extending into the lateral and third ventricles. The mass compressed the hypothalamus and suprasellar cistern while nearly obliterating the mammillo-pontine space. Imaging characteristics included T1 hypointensity, T2 hyperintensity, incomplete FLAIR suppression, and marked diffusion restriction on DWI (Fig. 4).

The surgical team employed a combined microscopic-endoscopic approach via interhemispheric craniotomy. The tumor had extensively infiltrated the corpus callosum, permitting interhemispheric visualization without callosotomy. Surgical findings revealed a pearly-white, well-encapsulated lesion displacing but not violating the pericallosal artery. Microsurgical resection addressed the main tumor mass, while endoscopic techniques managed ventricular extensions adherent to the right lateral ventricular wall and thalamostriate vein. A small portion of cyst wall was intentionally retained near critical venous structures to avoid complications.

Histopathology confirmed an epidermoid cyst. The patient recovered well postoperatively with resolution of intracranial hypertension symptoms, though preexisting memory deficits persisted. Financial limitations prevented postoperative MRI surveillance, with only 1 clinical follow-up documented.

### Case 3

A 54-year-old male presented with progressive 2-month symptoms of recurrent headaches, morning vomiting, and blurred vision, culminating in acute severe headache requiring emergency evaluation. Initial CT revealed a hypodense lateral ventricular lesion. Neurological exam showed isolated bilateral grade 2 papilledema.

**Fig. 5:**
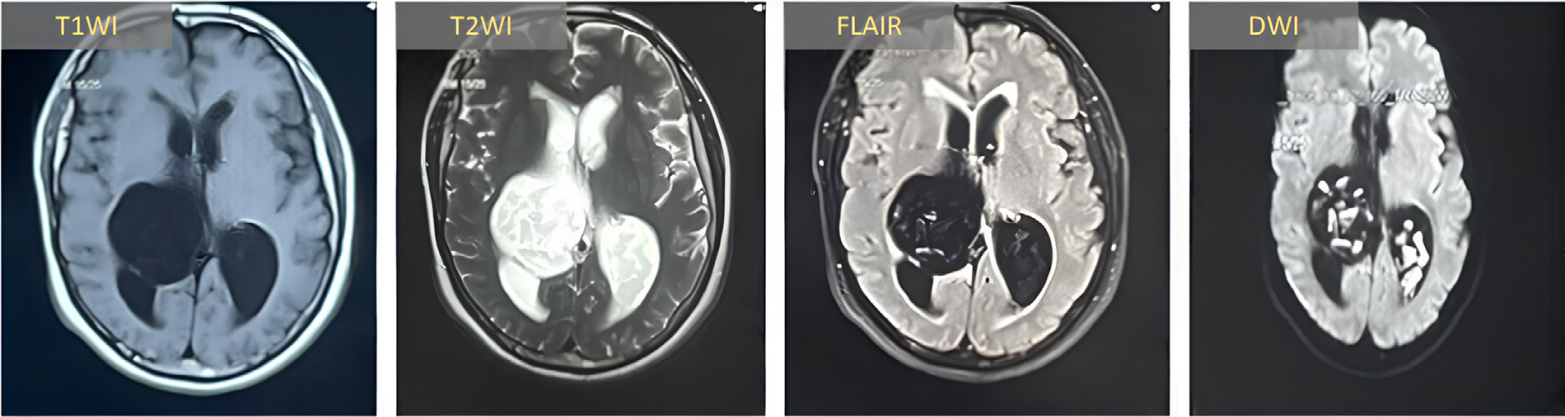
Right lateral ventricular epidermoid cyst with temporal and occipital horn extension. MRI reveals: (A) T1WI hypointensity; (B) T2WI hyperintensity; (C) incomplete FLAIR suppression; and (D) DWI restriction. The lesion demonstrates significant mass effect on the right thalamus with preservation of adjacent parenchymal architecture. *T1WI: T1-weighted imaging; T2WI: T2-weighted imaging; FLAIR: fluid-attenuated inversion recovery; DWI: diffusion-weighted imaging.*

MRI characterized a lobulated right ventricular mass extending into both temporal and occipital horns with contralateral occipital horn involvement, compressing the right thalamus. The lesion demonstrated classic epidermoid features: T1 hypointensity, T2 hyperintensity, incomplete FLAIR suppression, and DWI restriction (Fig. 5).

The surgical team performed a right trans-sulcal craniotomy using combined microsurgical and endoscopic techniques. The pearly-white, avascular capsule was meticulously dissected from arachnoid planes, with endoscopic assistance for ventricular components adherent to the thalamostriate vein. Complete resection achieved while preserving neurovascular integrity.

Histopathology confirmed epidermoid cyst diagnosis. The patient recovered without complications, showing complete symptomatic resolution at 2-week and 3-month clinical follow-ups, though financial constraints precluded postoperative imaging.

## Discussion

### Lateral ventricle epidermoid cysts: Clinical considerations

Lateral ventricle epidermoid cysts represent an exceptionally rare manifestation of an already uncommon pathological entity [5]. These lesions originate from ectopic embryonic epithelial cells during neural tube closure and demonstrate characteristically slow growth with benign histopathological features [6,7]. Their intraventricular location presents distinct diagnostic and surgical challenges compared to more frequent sites like the cerebellopontine angle or parasellar regions [[8], [9], [10]].

The deep anatomical position of the lateral ventricle contributes to the rarity of these lesions, which often remain clinically silent until achieving sufficient size to cause mass effect or CSF obstruction [11,12]. Symptomatology is typically nonspecific and dependent on lesion size and location. Headaches - observed in all 3 presented cases—frequently indicate elevated intracranial pressure. Other manifestations including hemiparesis, visual deficits, and memory impairment reflect either neurovascular compression or ventricular dysfunction [2,13].

Diagnostic challenges arise from the potential for radiological oversight [13]. However, the pathognomonic diffusion restriction on DWI reliably differentiates these lesions from other intraventricular pathologies such as arachnoid cysts [1,4,13]. This diagnostic hallmark proved critical in our case series for preoperative planning.

### Pathological and surgical characteristics

Epidermoid cysts exhibit well-demarcated capsules with clear boundaries, limited vascularity, gradual growth patterns, and loose internal architecture. Histologically, they contain caseous material from desquamated cells with frequent cholesterol crystal deposition [14]. While typically developing along basal cisterns while enveloping adjacent neurovascular structures, these lesions may infiltrate and disrupt normal brain architecture [15].

Complete surgical resection remains the therapeutic gold standard, with incomplete removal risking residual cyst secretion of inflammatory mediators that may provoke fever or CSF circulation abnormalities [1,16]. The deep ventricular location and proximity to critical structures like pericallosal arteries and thalamostriate veins frequently complicate total resection [2,5].

Endoscopic techniques prove particularly advantageous given the cysts' defined margins and avascular nature. Neuroendoscopy provides superior illumination and magnification for precise intervention while facilitating additional procedures like third ventricular floor fenestration to restore CSF dynamics [1,2]. Our series demonstrates that combined microscopic-endoscopic approaches effectively address surgical challenges, enabling safe resection while minimizing neurological risk.

### Clinical outcomes and management considerations

Meticulous surgical technique is paramount, particularly when cyst walls adhere to critical structures as seen in Cases 2 and 3. Strategic retention of residual wall components may be necessary to avoid iatrogenic injury. While potentially increasing recurrence risk, this compromise is justified by the lesions' indolent growth when balanced against patient safety [2,5].

Our series demonstrated favorable postoperative outcomes with substantial symptom resolution, though persistent visual deficits in 1 case underscore the potential for irreversible damage from prolonged compression. This reinforces the imperative for early diagnosis and intervention [1,5].

Long-term monitoring is crucial given recurrence rates approaching 33%, typically associated with incomplete resection [2]. Financial constraints preventing follow-up imaging in 2 of our cases highlight systemic barriers to comprehensive postoperative care.

### Broader implications and future directions

This series enriches the sparse literature on lateral ventricle epidermoid cysts, emphasizing the importance of advanced imaging, multidisciplinary surgical planning, and individualized management. Future research should establish standardized guidelines for diagnosis, surgical intervention, and long-term surveillance of these rare lesions.

## Key lessons and conclusions

1. Diagnostic imaging: DWI remains indispensable for accurate differentiation from other intraventricular pathologies and precise preoperative planning.
2. Surgical strategy: Combined microscopic-endoscopic techniques optimize visualization and access, facilitating safe resection of deep-seated lesions while preserving neurovascular integrity.
3. Individualized care: Each case underscores the necessity of tailoring surgical approaches to specific anatomical and clinical circumstances to achieve optimal outcomes.

### Study limitations

The small sample size and lack of long-term imaging follow-up in 2 cases due to financial constraints limit comprehensive assessment of recurrence patterns and extended outcomes. Presentation variability and surgical approach differences highlight the need for standardized management protocols.

### Clinical relevance

These findings provide valuable insights into managing this exceptionally rare neurosurgical entity. By documenting both successful outcomes and persistent challenges, this series serves as an important reference while emphasizing the need for multidisciplinary collaboration in diagnosis and treatment planning. Future efforts should focus on developing evidence-based protocols and addressing healthcare system barriers to ensure optimal longitudinal patient care.

## Author contributions

M.M. was responsible for the conception of the work, data collection, drafting the article, critical revisions, and obtaining approval of the final version of the manuscript. S.I., B.A. and M.A. contributed by drafting the article, and critical revisions. All authors read the final manuscript and were involved in direct patient care.

## Declaration

This study was conducted in accordance with the ethical standards of Nasser Institute Hospital's ethical committee, and no external funding was recieved for this study.

## Patient consent

The authors declare that they have obtained consent from the patient.
